# Risk for Pelvic Metastasis and Role of Pelvic Lymphadenectomy in Node-Positive Vulvar Cancer-Results from the AGO-VOP.2 QS Vulva Study

**DOI:** 10.3390/cancers14020418

**Published:** 2022-01-14

**Authors:** Linn Woelber, Monika Hampl, Christine zu Eulenburg, Katharina Prieske, Johanna Hambrecht, Sophie Fuerst, Ruediger Klapdor, Sabine Heublein, Paul Gass, Annika Rohner, Ulrich Canzler, Sven Becker, Mareike Bommert, Dirk Bauerschlag, Agnieszka Denecke, Lars Hanker, Ingo Runnebaumn, Dirk M. Forner, Fabienne Schochter, Maximilian Klar, Roxana Schwab, Melitta Koepke, Matthias Kalder, Peer Hantschmann, Dominik Ratiu, Dominik Denschlag, Willibald Schroeder, Benjamin Tuschy, Klaus Baumann, Alexander Mustea, Philipp Soergel, Holger Bronger, Gerd Bauerschmitz, Jens Kosse, Martin C. Koch, Atanas Ignatov, Jalid Sehouli, Christian Dannecker, Sven Mahner, Anna Jaeger

**Affiliations:** 1 Department of Gynecology and Gynecologic Oncology, University Medical Center Hamburg—Eppendorf, 20246 Hamburg, Germany; k.prieske@uke.de (K.P.); j.hambrecht@uke.de (J.H.); a.jaeger@uke.de (A.J.); 2 Colposcopy Center at the Jerusalem Hospital Hamburg, 20357 Hamburg, Germany; 3 Department of Gynecology, University Medical Center Duesseldorf, 40225 Duesseldorf, Germany; hampl@med.uni-duesseldorf.de; 4 Department of Epidemiology, UMCG, Universität Groningen, 9713 Groningen, The Netherlands; christine.eulenburg@googlemail.com; 5 Mildred Scheel Cancer Career Center HaTriCS4, University Medical Center Hamburg—Eppendorf, 20251 Hamburg, Germany; 6 Department of Obstetrics and Gynecology, University Hospital, LMU—University of Munich, 80377 Munich, Germany; sophie.fuerst@med.uni-muenchen.de (S.F.); sven.mahner@med.uni-muenchen.de (S.M.); 7 Department of Obstetrics and Gynecology, Hannover Medical School, 30625 Hannover, Germany; klapdor.ruediger@mh-hannover.de; 8 Department of Obstetrics and Gynecology, University Hospital Heidelberg, 69120 Heidelberg, Germany; sabine.heublein@med.uni-heidelberg.de; 9 Department of Gynecology and Obstetrics, University Hospital Erlangen, Comprehensive Cancer Center Erlangen-EMN, Friedrich-Alexander University Erlangen-Nuremberg, 91054 Erlangen, Germany; paul.gass@uk-erlangen.de; 10 Department of Gynecology, University Medical Center Tuebingen, 72076 Tuebingen, Germany; Annika.Rohner@med.uni-tuebingen.de; 11 Department of Gynecology and Obstetrics, University Hospital Dresden, Technische Universität Dresden, Dresden, Germany & National Center for Tumor Diseases (NCT), Partner Site Dresden, 01307 Dresden, Germany; ulrich.canzler@uniklinikum-dresden.de; 12 Department of Gynecology and Obstetrics, University Medical Center Frankfurt, 60590 Frankfurt, Germany; sven.becker@kgu.de; 13 Department of Gynecology and Gynecologic Oncology, Evang. Kliniken Essen-Mitte, 45136 Essen, Germany; m.bommert@kem-med.com; 14 Department of Gynecology, University Medical Center Kiel, 24105 Kiel, Germany; dirk.bauerschlag@uksh.de; 15 Department of Gynecology, Medical Center Wolfsburg, 38440 Wolfsburg, Germany; agnieszka.denecke@klinikum.wolfsburg.de; 16 Department of Gynecology and Gynecologic Oncology, University Medical Center Luebeck, 23562 Luebeck, Germany; lars.hanker@uksh.de; 17 Department of Gynecology, Jena University Hospital, 07743 Jena, Germany; Ingo.Runnebaum@med.uni-jena.de; 18 Department of Gynecology, Evangelisches Krankenhaus Kalk, 51103 Cologne, Germany; dirk.forner@evkk.de; 19 Department of Obstetrics and Gynecology, University of Ulm Medical Center, 89081 Ulm, Germany; fabienne.schochter@uniklinik-ulm.de; 20 Department of Gynecology, University Medical Center Freiburg, 79106 Freiburg, Germany; maximilian.klar@uniklinik-freiburg.de; 21 Department of Gynecology, University Medical Center Mainz, 55131 Mainz, Germany; Roxana.Schwab@unimedizin-mainz.de; 22 Department of Obstetrics and Gynaecology, University Hospital Augsburg, 86156 Augsburg, Germany; melitta.koepke@uk-augsburg.de (M.K.); christian.dannecker@med.uni-augsburg.de (C.D.); 23 Department of Gynecology and Obstetrics, University Hospital Marburg, 35043 Marburg, Germany; kalder@med.uni-marburg.de; 24 Department of Gynecology and Obstetrics, Medical Center Altoetting, 84503 Altoetting, Germany; p.hantschmann@innklinikum.de; 25 Department of Gynecology, University Medical Center Koeln, 50937 Koeln, Germany; dominik.ratiu@uk-koeln.de; 26 Department of Gynecology, Hochtaunuskliniken, 61352 Bad Homburg, Germany; dominik.denschlag@hochtaunus-kliniken.de; 27 Department of Gynecology, Medical Center Gynaecologicum Bremen, 28209 Bremen, Germany; prof.schroeder@gynaekologikum-bremen.de; 28 Department of Gynecology, University Medical Center Mannheim, 68167 Mannheim, Germany; benjamin.tuschy@umm.de; 29 Department of Gynecology, Medical Center Ludwigshafen, 67063 Ludwigshafen, Germany; baumann@klilu.de; 30 Department of Gynecology and Gynecologic Oncology, University Medical Center Bonn, 53127 Bonn, Germany; alexander.mustea@ukbonn.de; 31 Department of Gynecology, University Medical Center Minden, 32429 Minden, Germany; philipp.soergel@muehlenkreiskliniken.de; 32 Department of Gynecology and Obstetrics, Technical University of Munich, 81675 Munich, Germany; holger.bronger@tum.de; 33 Department of Gynecology, University Medical Center Goettingen, 37075 Goettingen, Germany; gerd.bauerschmitz@med.uni-goettingen.de; 34 Department of Gynecology, Sana Klinikum Offenbach, 63069 Offenbach am Main, Germany; jens.kosse@sana.de; 35 Department of Gynecology, Medical Center Ansbach, 91522 Ansbach, Germany; martin.koch@anregiomed.de; 36 Department of Obstetrics and Gynecology, University Hospital Magdeburg, 39120 Magdeburg, Germany; atanas.ignatov@med.ovgu.de; 37 Department of Gynecology with Center for Oncological SurgeryNOGGO e.V., Charite Universitatsmedizin Berlin, Corporate Member of Freie Universität Berlin, Humboldt-Universität zu Berlin, and Berlin Institute of Health, Virchow Campus Clinic, Charité Medical University, 13353 Berlin, Germany; jalid.sehouli@charite.de

**Keywords:** vulvar cancer, pelvic lymphadenectomy, radiotherapy, groin, recurrence, prognosis

## Abstract

**Simple Summary:**

In node-positive vulvar squamous cell cancer, questions of when and how to perform pelvic lymphadenectomy (LAE) as well as the optimal extent of pelvic treatment in general have been surrounded by considerable controversy. In Germany, systematic pelvic LAE is currently recommended as a staging procedure in patients at risk for pelvic nodal involvement in order to prevent morbidity caused by pelvic radiotherapy (RT) in patients without histologically-confirmed pelvic involvement. However, the population at risk for pelvic metastases remains insufficiently described, resulting in the potential overtreatment of a considerable proportion of patients with groin-positive disease. This applies to the indication to perform surgical staging but also to adjuvant RT of the pelvis without previous pelvic staging. Our study aims to describe the risk for pelvic lymph node metastasis with regard to positive groin nodes and to clarify the indication criteria for pelvic treatment in node-positive vulvar cancer.

**Abstract:**

The need for pelvic treatment in patients with node-positive vulvar cancer (VSCC) and the value of pelvic lymphadenectomy (LAE) as a staging procedure to plan adjuvant radiotherapy (RT) is controversial. In this retrospective, multicenter analysis, 306 patients with primary node-positive VSCC treated at 33 gynecologic oncology centers in Germany between 2017 and 2019 were analyzed. All patients received surgical staging of the groins; nodal status was as follows: 23.9% (73/306) pN1a, 23.5% (72/306) pN1b, 20.4% (62/306) pN2a/b, and 31.9% (97/306) pN2c/pN3. A total of 35.6% (109/306) received pelvic LAE; pelvic nodal involvement was observed in 18.5%. None of the patients with nodal status pN1a or pN1b and pelvic LAE showed pelvic nodal involvement. Taking only patients with nodal status ≥pN2a into account, the rate of pelvic involvement was 25%. In total, adjuvant RT was applied in 64.4% (197/306). Only half of the pelvic node-positive (N+) patients received adjuvant RT to the pelvis (50%, 10/20 patients); 41.9% (122/291 patients) experienced recurrent disease or died. In patients with histologically-confirmed pelvic metastases after LAE, distant recurrences were most frequently observed (7/20 recurrences). Conclusions: A relevant risk regarding pelvic nodal involvement was observed from nodal status pN2a and higher. Our data support the omission of pelvic treatment in patients with nodal status pN1a and pN1b.

## 1. Introduction

Despite its constantly increasing incidence (3–5/100,000/year in Europe) [1] and decreased age at onset, squamous cell vulvar cancer (VSCC) still remains a relatively rare disease, comprising approximately 5–6% of all gynecological malignancies [2]. Both disease-free survival (DFS) and overall survival (OS) are strongly dependent on inguinal lymph node involvement, as represented in 3-yearDFS rates of 35.2% and OS rates of 56.2% in node-positive patients vs. 3-year DFS rates of 75.2% and OS rates of 90.2% in node-negative disease [3]. Consequently, adjuvant treatment planning is based on groin status. Established clinical evidence with regard to pelvic nodal involvement and consecutive pelvic treatment is lacking. The overall risk for pelvic nodal involvement in previous small series ranges between 30% and 35% in node-positive VSCC and seems to rise with an increasing number of affected groin nodes [4,5,6,7,8]. In order to compare pelvic LAE with adjuvant radiotherapy, the Gynecologic Oncology Group (GOG) published a randomized trial conducted in the early eighties by Homesley et al.; patients with histologically-confirmed lymph node metastases to the groin after surgical groin dissection and vulvectomy received either irradiation to groins and pelvis with 45–50 Gy or pelvic LAE without any adjuvant radiotherapy (RT). Of 53 patients in the “pelvic LAE group”, 15 (28.3%) had histologically-positive pelvic nodes [9]. The study was stopped early due to a significant survival benefit in the “radiotherapy group”. Since publication of the study results, adjuvant irradiation of the groins and pelvis has been the standard approach to VSCC with >1 lymph node metastasis in the groin. However, the results of this study have to be critically discussed: 2-year OS was better in the “radiotherapy group” compared to the “pelvic LAE group” (68% vs. 54%), but the number of pelvic recurrences was higher in the radiotherapy group (6% vs. 2%). Most importantly, the poor outcome of the “pelvic LAE group” can mainly be attributed to the omission of adjuvant radiotherapy to the groins in this group, resulting in a groin recurrence rate of 23.6% compared to only 5.1% in the “radiotherapy group”. The prognosis in the case of groin recurrence is known to be unfavorable in the majority of cases with a 5-year OS of only 20% [10]. Therefore, the study fails to answer the question of the necessity of pelvic treatment at all or the benefits of a surgical staging in selected patients.

As irradiation of the pelvis can cause substantial morbidity, it is currently recommended by the German vulvar cancer guideline to perform a pelvic lymph node staging (laparoscopic or extraperitoneal) in patients at risk for nodal involvement of the pelvis in analogy to surgical lymph node staging in cervical cancer [11]. However, this approach often requires second surgery with increased morbidity, and the population at risk is very poorly defined [12]. According to the German guidelines, patients at risk are patients with one groin metastasis >5 mm and patients with >1 metastasis independent of size (including bilateral involvement) and metastasis with extracapsular spread [11]. These characteristics are known to be associated with a poor prognosis in general; however, whether this is due to pelvic involvement is unclear. If staging is not performed or pelvic nodal metastases are histologically confirmed, radiotherapy is recommended beyond radiotherapy to the affected groin.

The aim of the current study was to investigate the risk for pelvic lymph node metastasis with regard to positive groin nodes. Describing this relationship in more detail might help to prevent unnecessary pelvic treatment (surgery and radiotherapy) in a majority of groin node-positive patients.

## 2. Materials and Methods

### 2.1. Patients and Data Collection

This retrospective multicenter analysis was designed to collect therapeutic data from all patients diagnosed with primary groin node-positive VSCC after surgical staging of the groin/s between 2017 and 2019 at 33 gynecologic cancer centers in Germany that mainly already participated at the AGO-CaRE-1 study [3]. In total, *n* = 306 patients were included based on the above-mentioned inclusion criteria and analyzed with regard to pelvic treatment (pelvic LAE/no pelvic LAE and/or radiotherapy), and the risk for pelvic nodal involvement recurrent disease. Furthermore, the relation between groin and pelvic nodal involvement was investigated to better characterize the population at risk for pelvic nodal involvement. In addition, the impact of pelvic nodal metastases on prognosis and outcome was evaluated. Therefore, medical charts and pathological reports were reviewed. The study was approved by each local ethics committee (leading vote: Ethics Committee of the Medical Board Hamburg, Reference number: PV7431).

Data collection was based on an anonymized questionnaire and performed retrospectively from December 2020 until August 2021. For tumor documentation, TNM staging system Version 6 was used [13]. This study was performed by the AGO Study Group in collaboration with the AGO Kommission Vulva Vagina and the NOGGO led by the University Medical Center Hamburg–Eppendorf.

### 2.2. Statistical Analysis

Analysis was performed using Stata (StataCorp LLC, 4905 Lakeway Drive, College Station, TX, USA). Variables are described as median and range or count and percentage, respectively. Categorial variables are compared using Pearson’s chi square test or Fisher’s exact test, depending on the group sizes. Continuous variables were compared using the Mann–Whitney U-test. For survival data, Kaplan–Meier curves were displayed and comparisons calculated using the logrank test or Cox regression. All statistical tests were performed and interpreted using a level of significance of 5%. Disease-free survival (DFS) was calculated as the time interval between primary diagnosis and disease recurrence or death of any cause or censoring, and overall survival (OS) was the period resulting from primary diagnosis to death of any cause or censoring. Incidence rates and 1-year DFS and OS rates were reported instead of median survival rates, due to a relatively short follow-up. Comparison of patients with and without pelvic LAE regarding DFS and OS was performed twice: a comparison of originally observed data described the difference between the actual groups. A secondary propensity score analysis was performed to overcome the problem of imbalanced treatment groups using IPTW (inverse probability of treatment weighting) to mimic randomization and to decorrelate treatment from baseline characteristics (see Supplementary paragraph 1 and Figure S1). ROC analyses were performed to evaluate the prognostic performance of groin metastases to predict the existence of pelvic nodal metastases using the number of affected inguinal lymph nodes and the LNR per side (lymph node ratio: number of affected/number of excised nodes groin).

## 3. Results

### 3.1. Patients

A total of 306 patients with histologically-confirmed primary groin node-positive vulvar cancer was included in the study. Patient characteristics are summarized in Table 1. The majority had locally restricted tumors (pT1b/pT2; 95.4%; 292/306). The most common localization of the primary tumor was the medial anterior fourchette (36.3%; 111/306); 37/306 patients (12.1%) had no documented preoperative imaging (ultrasound/CT/MRI), while the remaining 269 patients (87.9%) received at least one of the three imaging procedures. A total of 52.2% of all patients who received preoperative imaging with documented results had suspicious findings in the groin, and in 4.5% of the cases, suspicious finding in the pelvis were detected. A total of 94.4% (289/306) underwent surgery as primary treatment for the primaries and tumor-free margins (R0 resection) were achieved in 89.2% (273/306). Groin staging was most often performed by bilateral inguino–femoral LAE (77.4%; 237/306). The median number of removed lymph nodes per groin was 7 (range 1–23), while the median number of affected lymph nodes was 1 (range 1–11). A total of 35.6% (109/306) received pelvic LAE as a staging procedure. A total of 70/109 (64.2%) patients received pelvic LAE during the same surgery as inguino–femoral LAE, and 39/109 (35.8%) patients received pelvic LAE as a second surgery. Of the 109 patients with pelvic LAE, 43/109 (39.4%) had unilateral and 66/109 (60.6%) bilateral LAE. A total of 19/66 (28.8%) patients with bilateral pelvic LAE had only unilateral lymph node involvement in the groin.

Pelvic nodal involvement was observed in 18.5% of the patients with pelvic LAE (20/108 patients, one patient with unknown status). The median number of removed pelvic lymph node per side was 9 (range 1–34) and the median number of affected pelvic lymph nodes in case of pelvic metastases was 2 (range 1–5). The median number of affected inguinal lymph nodes per groin within in the pelvic node-negative group was 1 compared to 2 in the pelvic node-positive group. No patient with nodal status pN1a or pN1b showed pelvic nodal involvement (see Table 2). Taking only patients with nodal status pN2a–c or pN3 and pelvic LAE (*n* = 80) into account, the rate of pelvic involvement was 25.0%.

In total, 64.4% (197/306) received adjuvant RT, while adjuvant chemoradiation was applied in 14.4% (47/306). The distribution of radiation fields with regard to pelvic nodal involvement and treatment are displayed in Table 3. Out of 161 patients at risk for pelvic involvement as for a nodal status pN2a and above, 126 (78.3%) received adjuvant radiotherapy, thereof only 34 (21.1%) including the pelvic field (17 patients, 10.6%, had unknown radiation fields). Furthermore, of the patients with histologically-confirmed pelvic nodal metastases, only 45% (9/20) received adjuvant treatment of the pelvis.

Surgery-related morbidity was assessed using four distinctive categories: wound breakdown, infection of the groins, need for re-surgery, and evidence of persistent lymphocele. In 3/4 categories, patients with pelvic LAE experienced (slightly) increased morbidity in comparison to patients without pelvic LAE (see Table 4).

### 3.2. Relation between Inguinal and Pelvic Nodal Involvement

Table 2 highlights the correlation between inguinal and pelvic nodal involvement. A relevant risk regarding pelvic metastasis was present from nodal status pN2a and higher. Receiver operating characteristics (ROC) analysis showed an AUC of 0.66 (95%CI: 0.52; 0.80) with 85.7% sensitivity and 30.2% specificity for the prediction of pelvic involvement in case of ≥2 positive groin nodes per patient. With regard to LNR, referring to the number of affected/the number of excised nodes per groin, the AUC was only 53.7%.

### 3.3. Recurrences

Follow-up was available in 291 patients. Within a median follow-up of 17.2 months (range 0.2–54.9 months), a total of 41.9% (122/291) patients experienced disease recurrence or death. Table 5 and Table 6 summarize the site of recurrence with regard to pelvic nodal status and treatment. As expected, the general risk for recurrence was higher in the pelvic node-positive cohort (60%, 12/20) compared to the pelvic node-negative subgroup (45.9%, 39/85 patients) and the pelvic status unknown group (31.7%, 59/186 patients).

Pelvic radiotherapy was associated with a reduced risk of recurrence and death in patients with confirmed metastases (see Table 7 and Table 8). In total, distant recurrences appeared as the most frequent site of recurrence (pelvic node-negative 18.8%, 16/85, pelvic node-positive 35%, 7/20) after this relatively short follow-up. Recurrences at the vulva occurred as the second most frequent site in the pelvic node-negative cohort (15.3%, 13/85), while the second most frequent site in the pelvic node-positive subgroup was pelvic recurrences (10%, 2/20).

### 3.4. Survival

Survival calculation was performed using propensity scoring and IPT weighting to mimic randomization and to decorrelate treatment from baseline characteristics (see Supplementary paragraph 2). The median DFS for all node-positive patients regardless of the pelvic nodal status was 18.7 months, while the median OS was not reached. As expected, in the case of pelvic nodal involvement, prognosis was impaired with a 1-year DFS of 19.8% (95%CI 3.7–45.2) versus 66.7% (95%CI 54.4–76.4) in the pelvic node-negative subgroup (HR: 3.50, 95%CI 1.81–6.77, *p*-value < 0.001). Table 7 and Table 8 display the outcome with regard to the applied pelvic treatment and pelvic nodal status.

**Figure 1:**
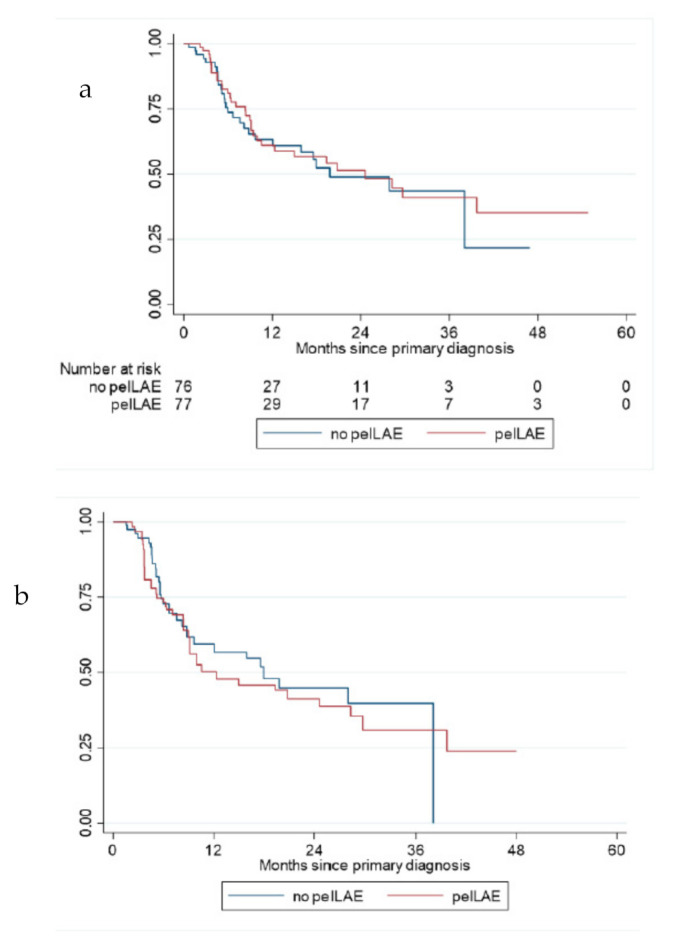
Kaplan–Meier curve for disease-free survival (DFS); local recurrences excluded, ≥pN2b (**a**) before IPTW and (**b**) after IPTW (inverse probability of treatment weighting). pelvLAE = pelvic lymphadenectomy.

In general, there was a trend towards a shorter 1-year DFS in patients undergoing pelvic LAE in comparison to the patients without pelvic LAE (1-year DFS: 58.9% versus 70.1%; HR: 1.39, 95%CI 0.96; 2.01, *p* = 0.080). However, after IPT weighting and exclusion of patients with a nodal status pN1a/b, the trend towards worse prognosis in the pelvic LAE subgroup was no longer visible (1 year. survival rate: 50.8% versus 46.4%; HR: 1.10, 95%CI 0.67–1.78, *p*-value 0.713, see Figure 1a,b).

## 4. Discussion

The role of pelvic LAE in node-positive VSCC is a matter of discussion, as the population at risk for pelvic nodal involvement remains poorly described. As a consequence, the question when and how to reasonably recommend pelvic treatment in general has been insufficiently answered. The present analysis focuses on 306 well-characterized node-positive patients with regard to risk of pelvic metastasis and the impact of pelvic involvement on prognosis and outcome of affected patients.

In our study, pelvic nodal involvement was observed in only 18.5% of all node-positive patients. Furthermore, a relevant risk for pelvic metastasis was only present from nodal status pN2a and higher. None of the patients with one or two metastases <5 mm or one intranodal metastasis ≥5 mm showed pelvic nodal involvement. Taking only the patients with increased risk for pelvic metastases into account (nodal status ≥pN2a), the risk for pelvic nodal involvement was still lower than expected with 25%. According to previously published series, the expected risk for pelvic metastasis ranges between 30 and 35% in node-positive VSCC [9,14,15,16]. This discrepancy might be caused by an overestimation of the percentage of pelvic metastasis due to a negative selection bias in earlier series, as the decision for pelvic LAE was often made on an individual basis. Our results suggest that pelvic treatment is justified only for patients with a nodal status pN2a or higher; importantly, this also applies to adjuvant RT to the pelvis. Even then 75% of node-positive patients with nodal status ≥pN2a might need neither pelvic surgical staging nor RT. Unfortunately, further narrowing of the population at risk was not possible with the current dataset. Neither the number of positive nodes alone nor the lymph node ratio were suitable to sufficiently describe the relation between nodal involvement of the groin and the pelvis. Probably the number as well as the size of affected groin lymph nodes are relevant with regard to the risk of pelvic metastasis.

Currently, international treatment recommendations are very heterogeneous with regard to adjuvant treatment in node-positive VSCC. This is due to the fact that the prognostic impact of the number of affected lymph nodes and the subsequent benefit of irradiation are controversial [17,18]. However, all guidelines recommend adjuvant RT to the pelvic field if adjuvant RT to the groins is indicated, and no surgical staging of the pelvic nodes is performed. While the European Society of Gynecological Oncology (ESGO) guideline recommends adjuvant RT in patients with more than one metastatic lymph node and/or presence of extracapsular spread [19], the German guideline advises adjuvant RT in patients with one groin metastasis >5 mm, in patients with ≥2 metastases independent of size (including bilateral involvement) [11]. The NCCN guideline even recommends adjuvant treatment to the groins and pelvis already in patients with one metastasis >2 mm [20]. Based on our results this approach seems to be no longer justified. Our data suggest that the risk of pelvic metastases is extremely low in patients with only one intracapsular metastasis and two metastasis <5 mm (pN1a/b). This supports the omission of pelvic radiotherapy (and pelvic surgical staging) in these patients.

As mentioned above, prognosis in VSCC is mainly determined by nodal status and is impaired for groin node-positive patients irrespective of the pelvic nodal status. However, in case of pelvic nodal involvement, outcome is even more unfavorable with a 1-year-survival rate of only 19.8% compared to 66.7% in the pelvic node-negative subgroup of our cohort. Generally, adjuvant RT is applied to prevent recurrences as well as to subsequently improve patients’ outcomes. In our study, any kind of adjuvant RT was applied to 78.3% of all patients potentially at risk for pelvic metastasis; only 21% of them received RT including the pelvic field. Furthermore, in the case of histologically-confirmed pelvic metastasis, only 45% received adjuvant RT to the pelvis. The reasons for the insufficient performance of RT were not documented in this study. Most likely co-morbidities and surgical side-effects such as wound breakdown (52.3%) can be held responsible. The general risk reducing effect of radiotherapy in the case of pelvic nodal involvement was confirmed by our study with an incidence of a DFS event in 10 person-years of 8.9 vs. 13.3 with and without adjuvant RT to the pelvic field, respectively. Nevertheless, pelvic recurrences were not the leading problem in patients at risk for pelvic nodal metastases; in our study 41.9% of the patients experienced any kind of recurrence or death within a median follow-up of 17.2 months. Distant recurrences appeared as the most frequent site (pelvic node-negative 19.8%, pelvic node-positive 35%). Isolated pelvic recurrences occurred in 4.8% of the patients at risk for pelvic metastasis (≥pN2a) without pelvic staging and 2.4% of the surgically staged pelvic N– group compared to 10% of the pelvic N+ group (45% of whom received adjuvant RT to the pelvic region). These results have to be interpreted in view of the relatively short FU of 17.2 months. In case of a longer FU duration, an increased rate of local recurrences would have been expected, as the overall local recurrence rate even in patients with early-stage VSCC is reported to remain high with 27.2% at 5 years and 39.5% at 10 years without plateauing [21]. The duration of FU was considered to be appropriate for our analysis, focusing on node-positive disease as nodal and distant recurrences occur early in the course of the disease [22]. In view of the high risk for distant recurrences and the poor prognosis, it remains questionable whether the investigated treatment approaches are the right choices at all to improve the outcome in patients with nodal metastases and especially pelvic metastases. An improvement in systemic treatment beyond RT in high risk patients should be investigated more intensively in the future. The addition of chemotherapy to RT can only be a small step into this direction. Like in other entities, immuno-oncology might change the field [23,24].

When discussing the optimal extent of pelvic treatment, also the side effects and potential complications of the treatment options should be taken into consideration. Both pelvic LAE and RT are known to be associated with increased morbidity and decreased quality of life. In our study, patients with pelvic LAE experienced increased surgical-related morbidity compared to patients without pelvic LAE. These results are in line with previously published data. Herein, major complications were observed to be twice as common in patients treated with pelvic LAE compared to patients with groin dissection only [25]. In addition to the well-known surgery-related side effects, it is particularly noteworthy that a relevant number of patients (approximately 35%) did not receive adjuvant RT at all due to various reasons and therefore experienced worse outcome. In view of the relatively low incidence of pelvic nodal involvement, the high surgery-related morbidity and especially the poor prognosis, pelvic LAE as a staging procedure should be applied more restrictively in the future in order to prevent unnecessary harm to the majority of mostly older patients. The same applies to pelvic RT, which is also known to be associated with considerable toxicity such as genitourinary reaction, sexual dysfunction, skin desquamation and edema in the lower extremity [17,26].

## 5. Conclusions

Pelvic metastasis occurred in 18.5% of node-positive VSCC. A relevant risk for pelvic nodal involvement was only observed from nodal status pN2a and higher. This supports the omission of pelvic radiotherapy and pelvic surgical staging in patients with only one intracapsular metastasis or ≤2 metastases <5 mm. Beyond the nodal status, no sufficient predictive model for the involvement of the pelvic nodes could be determined. Moreover, half of the surgically staged and pelvic node-positive patients did not receive adjuvant RT to the pelvis and therewith experienced a worse outcome. Taking the poor prognosis in cases of pelvic nodal involvement and the high risk for distant recurrences in this group into account, additional therapeutic approaches beyond surgery and RT such as systemic therapies must be evaluated for high risk disease in the future.

## Figures and Tables

**Table 1:** Patient characteristics (*n* = 306) with regard to surgical approach.

Characteristics	Total *n* = 306	Pelvic LAE No *n* = 197 (64.4%)	Pelvic LAE Yes *n* = 109 (35.6%)	*p*-Value *
Tumor stage				0.768 ^b^
pT1b	259 (84.6%)	168 (85.3%)	91 (83.5%)	
pT2	33 (10.8%)	22 (11.2%)	11 (10.1%)	
pT3	6 (1.9%)	3 (1.5%)	3 (2.8%)	
T4	2 (0.7%)	1 (0.5%)	1 (0.8%)	
unknown	6 (2%)	3 (1.5%)	3 (2.8%)	
BMI median (range)	26 (15–45)	25.5 (15–45)	27.1 (18–45)	0.195 ^a^
ECOG Status				0.388 ^c^
ECOG 0	89 (29.1%)	56 (28.4%)	33 (30.3%)	
ECOG 1	87 (28.4%)	62 (31.5%)	25 (22.9%)	
ECOG 2	26 (8.5%)	20 (10.2%)	6 (5.5%)	
ECOG 3+4	7 (2.3%)	6 (3%)	1 (0.9%)	
unknown	97 (31.7%)	53 (26.9%)	44 (40.4%)	
Median tumor diameter (mm) (range)	32 (2–110)	32.5 (2–106)	32 (4–110)	0.863 ^a^
Median depth of invasion (mm) (range)	7 (1–120)	7 (1–120)	7 (1–65)	0.280 ^a^
Resection status of vulvar primary				0.225 ^b^
R0	273 (89.2%)	172 (87.3%)	101 (92.6%)	
R1	21 (6.9%)	16 (8.1%)	5 (4.6%)	
unknown	12 (3.9%)	9 (4.6%)	3 (2.8%)	
Median minimal resection margin (mm) (range)	3.0 (0–22)	3.0 (0–22)	3.25 (0–15)	0.970 ^a^
Nodal status groin				<0.001 ^b^
pN1a	73 (23.9%)	66 (33.5%)	7 (6.4%)	
with one metastasis <5 mm	53 (17.3%)	49 (24.9%)	4 (3.6%)	
with two metastasis <5 mm	20 (6.6%)	17 (8.6%)	3 (2.8%)	
pN1b	72 (23.5%)	51 (25.9%)	21 (19.3%)	
pN2a/b	62 (20.2%)	34 (17.3%)	28 (25.7%)	
pN2c/pN3	97 (31.7%)	44 (22.3%)	53 (48.6%)	
unknown	2 (0.7%)	2 (1.0%)	0	
Median number of removed LN groin (range)	7 (1–23)	6 (1–21)	7 (1–23)	0.003 ^a^
Median number of affected LN groin (range)	1 (1–11)	1 (1–7)	1 (1–11)	0.065 ^a^
Median number of removed SN LN groin (range)	2 (1–11)	2 (1–11)	2 (1–10)	0.990 ^a^
Median number of affected SN LN groin (range)	1 (1–6)	1 (1–6)	1 (1–4)	0.141 ^a^
Median number of removed LN pelvis (range)	9 (1–34)	n.a.	9 (1–34)	n.a.
Median number of affected LN pelvis (range)	2 (1–5)	n.a.	2 (1–5)	n.a.
Distant metastasis				<0.001 ^b^
M0	256 (83.6%)	178 (90.4%)	78 (71.6%)	
M1	32 (10.5%)	8 (4%)	24 (22%)	
unknown	18 (5.9%)	11 (5.6%)	7 (6.4%)	
Treatment of the primary				0.346 ^b^
surgery	289 (94.4%)	189 (95.9%)	100 (91.7%)	
no surgical treatment (radiotherapy or chemoradiation)	14 (4.6%)	6 (3%)	8 (7.3%)	
(radiotherapy or chemoradiation)				
unknown	3 (1%)	2 (0.1%)	1 (0.9%)	
Surgical therapy vulva				0.832 ^b^
complete vulvectomy	75 (24.5%)	48 (24.4%)	27 (24.7%)	
partial vulvectomy	183 (59.8%)	119 (60.4%)	64 (58.7%)	
wide excision	35 (11.4%)	22 (11.2%)	13 (12%)	
exenteration	3 (1%)	1 (0.5%)	2 (1.8%)	
unknown	10 (3.3%)	7 (3.5%)	3 (2.8%)	
SNL—LAE groin				0.012 ^b^
unilateral	18 (5.9%)	14 (7.1%)	4 (3.6%)	
bilateral	137 (44.8%)	98 (49.7%)	39 (35.8%)	
no SNL-LAE groin	151 (49.3%)	85 (43.2%)	66 (60.6%)	
Inguino-femoral LAE				0.001 ^b^
unilateral	49 (16%)	38 (19.3%)	11 (1.1%)	
bilateral	237 (77.4%)	140 (71.1%)	97 (89%)	
no inguino-femoral LAE	20 (6.6%)	19 (9.6%)	1 (0.9%)	
Pelvic LAE				n.a.
unilateral	43 (14.1%)	0	43 (39.4%)	
bilateral	66 (21.5%)	0	66 (60.6%)	
no pelvic LAE	197 (64.4%)	197 (100%)	0	
Surgical approach pelvic LAE				n.a.
laparascopy	67 (21.9%)	0	67 (61.4%)	
extraperitoneal via groin	35 (11.4%)	0	35 (32.1%)	
open	6 (2%)	0	6 (5.6%)	
unknown	198 (64.7%)	197	1 (0.9%)	
Adjuvant radiotherapy				0.001^b^
yes	197 (64.4%)	115 (58.3%)	82 (75.2%)	
no	103 (33.7%)	80 (40.6%)	23 (21.1%)	
unknown	6 (1.9%)	2 (0.1%)	4 (3.7%)	
Adjuvant chemoradiation				
yes	47 (15.4%)	20 (10.2%)	27 (24.8%)	
no	256 (83.7%)	176 (89.3%)	80 (73.4%)	
unknown	3 (0.9%)	1 (0.5%)	2 (1.8%)	
Primary chemoradiation				0.085 ^b^
yes	14 (4.6%)	6 (3%)	8 (7.3%)	
no	289 (94.4%)	189 (95.9%)	100 (91.7%)	
unknown	3 (1%)	2 (0.1%)	1 (0.9%)	
Median total dose applied (Gy) (range)	50.4 (2–70)	50.4 (2–66)	50.4 (26–70)	0.332 ^a^
Recurrent disease				0.079 ^d^
yes	102 (33.3%)	54 (27.4%)	48 (44%)	
no	181 (59.2%)	125 (63.4%)	56 (51.4%)	
unknown	11 (3.6%)	8 (4.1%)	3 (2.8%)	
death without relapse	12 (3.9%)	10 (5.1%)	2 (1.8%)	

*p-value for the comparison no pelvic LAE vs. pelvic LAE from ^a^ Mann–Whitney U-test, ^b^ Pearson chi-square test, ^c^ Fisher’s exact test, and ^d^ logrank test. pT3/4 categories were combined for statistical testing. Missing/unknown categories were excluded for statistical testing. *p*-value < 0.05 was considered as statistically significant. N.a.: not applicable.

**Table 2:** Relation between inguinal and pelvic nodal status (*n* = 108 patients with known pelvic status).

Nodal Status Groin	Total *n* = 108	Nodal Status Pelvis N0 (Pelvic N−) *n* = 88	Nodal Status Pelvis N+ (Pelvic N+) *n* = 20	*p*-Value *
				0.001
pN1a	7 (6.5%)	7 (8.0%)	0	
pN1b	21 (19.4%)	21 (23.9%)	0	
pN2a/b	28 (25.9%)	26 (29.5%)	2 (10.0%)	
pN2c/pN3	52 (48.2%)	34 (38.6%)	18 (90.0%)	

*p-value for the relation between inguinal and pelvic nodal status from Fisher’s exact test. *p*-value <0.05 was considered as statistically significant.

**Table 3:** Adjuvant radiotherapy (RT) with regard to pelvic treatment and pelvic nodal status.

Radiation Fields	Total *n* = 305	No Pelvic LAE *n* = 197	Pelvic LAE Nodal Status Pelvis N0 (Pelvic N−) *n* = 88	Pelvic LAE Nodal Status Pelvis N1 (Pelvic N+) *n* = 20
No groin RT (including vulva only)	99 (32.4%)	81 (41.1%)	12 (13.6%)	6 (30.0%)
Groins+/− vulva	125 (41.0%)	70 (35.6%)	51 (58.0%)	4 (20.0%)
Groins + pelvis +− vulva	50 (16.4%)	31 (15.7%)	10 (11.3%)	9 (45.0%)
pelvis only	3 (1%)	3 (1.5%)	0	0
Missing	28 (9.2%)	12 (6.1%)	15 (17.1%)	1 (5.0%)

*n* = 305, 1 patient with unknown pelvic status omitted.

**Table 4:** Complications with reagrd to surgical approach.

Complications	Total *n* = 306	Pelvic LAE No *n* = 197 (64.4%)	Pelvic LAE Yes *n* = 109 (35.6%)	*p*-Value *
Woundhealing problems postoperatively				0.930
yes	89 (29.0%)	57 (29.0%)	32 (29.4%)	
no	192 (62.8%)	124 (62.9%)	68 (62.3%)	
unknown	25 (8.2%)	16 (8.1%)	9 (8.3%)	
Infection of the groin				0.025
yes	77 (25.1%)	41 (20.8%)	36 (33%)	
no	204 (66.7%)	138 (70%)	66 (60.6%)	
unknown	25 (8.2%)	18 (9.2%)	7 (6.4%)	
Secondary surgery needed				0.341
yes	80 (26.1%)	48 (24.4%)	32 (29.3%)	
no	226 (73.9%)	149 (75.6%)	77 (70.7%)	
Persistent lymphocele				0.223
yes	61 (19.9%)	36 (18.3%)	25 (22.9%)	
no	197 (64.4%)	133 (67.5%)	64 (58.7%)	
missing	48 (15.7%)	28 (14.2%)	20 (18.4%)	

*p-value for the comparison of complications with regard to surgical approach via Pearson chi-square test. Missing categories were excluded for statistical testing. *p*–value < 0.05 was considered as statistically significant.

**Table 5:** Site of recurrence with regard to pelvic nodal status and available FU data.

Site of Recurrence	Total *n* = 291 *	No Pelvic LAE *n* = 186	Pelvic LAE Nodal Status Pelvis N0 (Pelvic N−) *n* = 85	Pelvic LAE Nodal Status Pelvis N1 (Pelvic N+) *n* = 20
No recurrence	169 (58.1%)	117 (62.9%)	45 (52.9%)	7 (35.0%)
Local (Vulva only)	34 (11.7%)	20 (10.7%)	12 (15.3%)	1 (5.0%)
Groins only	14 (4.8%)	10 (5.3%)	3 (3.5%)	1 (5.0%)
Vulva + groins	4 (1.4%)	4 (2.1%)	0	0
Pelvis only	9 (3.1%)	6 (3.2%)	2 (2.4%)	1 (5.0%)
Including pelvis	5 (1.7%)	1 (0.5%)	2 (2.4%)	2 (10.0%)
Including distant	35 (12.0%)	12 (6.5%)	16 (18.8%)	7 (35.0%)
Unknown location	10 (2.4%)	7 (3.7%)	3 (3.5%)	0
Death without relapse	11 (3.8%)	10 (5.1%)	1 (1.2%)	1 (5.0%)

* Follow-up data available in 291 patients.

**Table 6:** Pelvic recurrences with regard to radiation field.

Site of Recurrence	Total *n* = 265 *	No Pelvic LAE RT Including Pelvis (Yes) *n* = 142	No Pelvic LAE RT Including Pelvis (No) *n* = 33	Pelvic LAE (Pelvic N+) RT Including Pelvis (Yes) *n* = 9	Pelvic LAE (Pelvic N+) RT Including Pelvis (No) *n* = 10	Pelvic LAE (Pelvic N−) RT Including Pelvis (Yes) *n* = 10	Pelvic LAE (Pelvic N−) RT Including Pelvis (No) *n* = 61
Including pelvis	14 (5.3%)	5 (3.5%)	2 (6.1%)	0	3 (30.0%)	1 (10.0%)	3 (4.9%)
Including distant	33 (12.4%)	11 (7.7%)	1 (3.0%)	5 (55.5%)	2 (20.0%)	2 (20.0%)	12 (19.7%)
Unknown	7 (2.6%)	6 (4.2%)	0	0	0	0	1 (1.6%)
Total	54 (20.4%)	22 (15.4%)	3 (9.1%)	5 (55.5%)	5 (50.0%)	3 (30.0%)	16 (26.2%)

* Data available in 265 patients with available FU (*n* = 291) and with known radiation fields and either without pelvic LAE or known pelvic status after surgery.

**Table 7:** Prognosis with regard to surgical approach.

	Total ^#^ *n* = 291	Pelvic LAE No *n* = 86 (64.4%)	Pelvic LAE Yes *n* = 105 (35.6%)	*p*-Value *
1year DFS in % (95% CI)	65.9 (59.3–71.6)	70.1 (61.9–76.9)	58.9 (47.7–68.5)	0.079
1year OS in % (95% CI)	86.6 (81.3–90.6)	90.2 (83.9–94.1)	80.7 (69.8–87.9)	0.100

^#^ Follow-up data available in 291 patients. DFS = disease-free survival; OS = overall survival. * *p*-value for the prognosis with regard to surgical approach via logrank test. *p*-value < 0.05 was considered as statistically significant.

**Table 8:** Prognosis with regard to applied pelvic radiotherapy, pelvic nodal status, and surgical approach.

	Total ^#^ *n* = 265	No Pelvic LAE Rt Including Pelvis (Yes) *n* = 142	No Pelvic LAE Rt Including Pelvis (No) *n* = 33	Pelvic LAE (Pelvic N+) Rt Including Pelvis (Yes) *n* = 9	Pelvic LAE (Pelvic N+) Rt Including Pelvis (No) *n* = 10	Pelvic LAE (Pelvic N−) Rt Including Pelvis (Yes and No) *n* = 19	Pelvic LAE (Pelvic N−) Rt Including Pelvis (Yes) *n* = 10	Pelvic LAE (Pelvic n-) Rt Including Pelvis (No) *n* = 61	Pelvic LAE (Pelvic N−) Rt Including Pelvis (Yes and No) *n* = 71
1year DFS in % (95% CI)		67.7 (58.2–75.6)	78.8 (58.3–90.0)	43.8 (10.1–74.2)	11.1 (0.6–38.8)	19.8 (3.7–45.2)	45.0 (13.8–72.4)	69.2 (47.1–72.1)	65.4 (51.8–76.0)
Incidence of a DFS event in 10 person-years		2.06	3.04	8.88	13.19	9.1	3.81	3.19	3.29
Incidence of an OS event in 10 person-years		1.27	0.21	3.81	7.26	4.5	2.21	0.99	1.17

^#^ 38 patients with missing follow-up or missing radiation site were omitted. RT = radiotherapy; DFS = disease-free survival; OS = overall survival.

## Data Availability

Restrictions apply to the availability of these data. Data were obtained from the AGO study group and can be made available for subprojects after application at office-wiesbaden@ago-ovar.de.

## Supplementary Materials

The following is available online at https://www.mdpi.com/article/10.3390/cancers14020418/s1, Figure S1: Propensity scoring for pelvic LAE before (blue) and after (red) IPTW (≥pN2a), Supplementary paragraph 1: Additional explanation of Section 2.2. Statistical Analysis, Supplementary paragraph 2: Additional explanation of Section 3.4. Survival.
