# Supplementary material for: Risk for Pelvic Metastasis and Role of Pelvic Lymphadenectomy in Node-Positive Vulvar Cancer-Results from the AGO-VOP.2 QS Vulva Study
Source: Cancers (Basel). 2022 Jan 14;14(2):418. doi: 10.3390/cancers14020418 (PMC8773532; doi:10.3390/cancers14020418)
Supplement: Supplementary file 1 [file cancers-14-00418-s001.zip › cancers-1525466-supplementary.pdf]

cancers

MDPI

Supplementary Material: Risk for Pelvic Metastasis and Role of Pelvic Lymphadenectomy in Node-Positive Vulvar Cancer-Results from the AGO-VOP.2 QS Vulva Study

Linn Woelber *, Monika Hampl, Christine zu Eulenburg, Katharina Prieske, Johanna Hambrecht, Sophie Fuerst, Ruediger Klapdor, Sabine Heublein, Paul Gass, Annika Rohner, Ulrich Canzler, Sven Becker, Mareike Bommert, Dirk Bauerschlag, Agnieszka Denecke, Lars Hanker, Ingo Runnebaumn, Dirk M. Forner , Fabienne Schochter, Maximilian Klar, Roxana Schwab, Melitta Koepke, Matthias Kalder, Peer Hantschmann, Dominik Ratiu, Dominik Denschlag, Willibald Schroeder, Benjamin Tuschy, Klaus Baumann, Alexander Mustea, Philipp Soergel, Holger Bronger, Gerd Bauerschmitz, Jens Kosse, Martin C. Koch, Atanas Ignatov, Jalid Sehouli, Christian Dannecker, Sven Mahner and Anna Jaeger

Supplementary paragraph 1

2.2. Statistical Analysis

Propensity Score Computation

The best evidence for the comparison of treatment options results from randomized controlled trials. Since randomization was not performed in this study, but in contrast, treatment was administered with respect to certain clinical characteristics, a statistical propensity score technique was applied to mimic randomization and to un-correlate treatment from baseline characteristics. Inverse-probability-of-treatment-weighting (IPTW) is an established method to estimate causal treatment effects from observational data [16]. In short, individual patient data is weighed inverse-proportionally to the patient's likelihood to receive the treatment. This technique was applied to achieve balance regarding the baseline variables across the treatment groups (see Figure S1). Variables included in the IPTW algorithm were pT (1b, 2, 3/4), pN (1a vs >1a), R0 vs. R1, adjuvant treatment (yes vs. no), and radiation (no radiation and vulva only, groins +/– vulva, including pelvis, missing location).

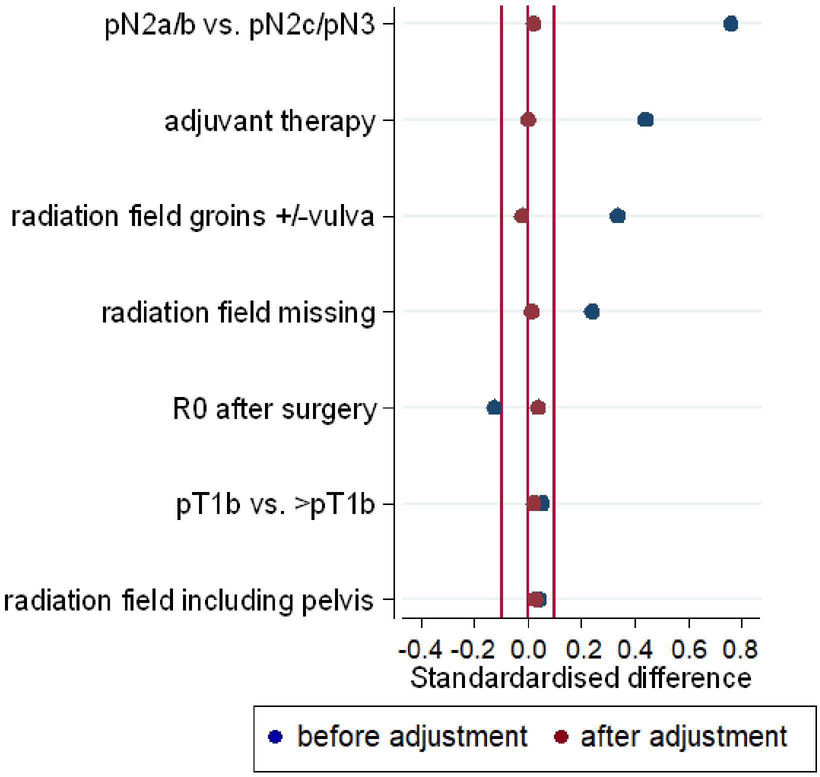

Figure S1. Propensity scoring for pelvic LAE before (blue) and after (red) IPTW (≥pN2a).

Supplementary paragraph 2

3.4. Survival

Survival calculations were performed twice: first on the originally observed data, to display the prognosis in the two treatment groups. In a second step, a propensity score analysis was performed to mimic randomization across LAE subgroups. A propensity score was calculated with respect to the variables pT (1b, 2, 3/4), pN (1a vs >1a), R0 vs. R1, adjuvant treatment (yes vs. no), and radiation (no radiation and vulva only, groins +/– vulva, including pelvis, missing location. The subjects were weighed in the Cox regression model, according to the inverse propensity score (IPTW). This technique induced balance across LAE subgroups with respect to these variables, expressed as standardized differences <0.1 (see Figure 1a,b).

Reference

16. Woelber, L.; Bommert, M.; Prieske, K.; Fischer, I.; Zu Eulenburg, C.; Vettorazzi, E.; Harter, P.; Jueckstock, J.; Hilpert, F.; de Gregorio, N.; et al. Pelvic Lymphadenectomy in Vulvar Cancer—Does it make sense? *Geburtshilfe Frauenheilkd.* **2020**, *80*, 1221–1228. https://doi.org/10.1055/a-1120-0138.
